# Genome-wide DNA methylation analysis in Chinese Chenghua and Yorkshire pigs

**DOI:** 10.1186/s12863-021-00977-0

**Published:** 2021-06-16

**Authors:** Kai Wang, Pingxian Wu, Shujie Wang, Xiang Ji, Dong Chen, Anan Jiang, Weihang Xiao, Yiren Gu, Yanzhi Jiang, Yangshuang Zeng, Xu Xu, Xuewei Li, Guoqing Tang

**Affiliations:** 1 Farm Animal Genetic Resources Exploration and Innovation Key Laboratory of Sichuan Province, Sichuan Agricultural University, Chengdu, China; 2 Sichuan Animal Science Academy, Chengdu, 610066 China; 3 College of Life Science, Sichuan Agricultural University, Yaan, China; 4 Sichuan Animal Husbandry Station, Chengdu, 610041 China

**Keywords:** DNA methylation, RRBS, Chenghua pig, Yorkshire pig

## Abstract

**Background:**

The Chinese Chenghua pig (CHP) is a typical Chinese domestic fatty pig breed with superior meat quality characteristics, while the Yorkshire pig (YP) has the characteristics of fast growth and a high rate of lean meat. Long term natural selection and artificial selection resulted in great phenotypic differences between the two breeds, including growth, development, production performance, meat quality, and coat color. However, genome-wide DNA methylation differences between CHP and YP remain unclear.

**Results:**

DNA methylation data were generated for muscle tissues of CHP and YP using reduced representation bisulfite sequencing (RRBS). In this study, a total of 2,416,211 CpG sites were identified. Besides, the genome-wide DNA methylation analysis revealed 722 differentially methylated regions (DMRs) and 466 differentially methylated genes (DMGs) in pairwise CHP vs. YP comparison. Six key genomic regions (*Sus scrofa* chromosome (SSC)1:253.47–274.23 Mb, SSC6:148.71–169.49 Mb, SSC7:0.25–9.86 Mb, SSC12:43.06–61.49 Mb, SSC14:126.43–140.95 Mb, and SSC18:49.17–54.54 Mb) containing multiple DMRs were identified, and differences of methylation patterns in these regions may be related to phenotypic differences between CHP and YP. Based on the functional analysis of DMGs, 8 DMGs (*ADCY1*, *AGBL4*, *EXOC2*, *FUBP3, PAPPA2*, *PIK3R1*, *MGMT* and *MYH8*) were considered as important candidate genes associated with muscle development and meat quality traits in pigs.

**Conclusions:**

This study explored the difference in meat quality between CHP and YP from the epigenetic point of view, which has important reference significance for the local pork industry and pork food processing.

**Supplementary Information:**

The online version contains supplementary material available at 10.1186/s12863-021-00977-0.

## Background

Epigenetic modifications of the genome can have both short-term and long-term effects on gene expression in different environments [1]. In turn, changes in these expression profiles have implications for multiple traits. DNA methylation was the first discovered epigenetic modification and one of the most thoroughly studied [2]. DNA methylation predominantly occurs at the C-5 position of cytosine in cytosine and guanine dinucleotide (CpG) dinucleotides in mammals [3]. Moreover, DNA methylation is critical for mammalian growth and development [4]. DNA methylation is traditionally regarded as a heritable and stable silence marker, which is essential for X-inactivation [5], silencing of genomic elements such as transposons [6], and genetic imprinting [7]. In addition, variation in DNA methylation involves in a wide range of cellular functions and pathologies [1], and DNA methylation also affects muscle growth and development [3]. Recently, the role of DNA methylation dynamics on skeletal muscle development and disease has been reported [8].

As the main meat source and human medical research model [9], the pig has important research value. Long-term domestication and modern breeding have resulted in both genetic variation and epigenetic modification in different breeds in pigs. Yorkshire pig (YP) is an important commercial pig breed with a high growth rate and lean meat [10]. Chenghua pig (CHP) is a Chinese local breed which is famous for superior meat quality [11]. By contrast, there are significant differences in body composition, muscle, and fat content between Chinese local pigs and commercial pigs [12], especially between CHP and YP [13]. Epigenetic variations, and in particular DNA methylation, might not only influence differences between individuals but also between populations [14]. Hence, DNA methylation might contribute to phenotype variation between pig breeds.

Recently, some studies have explored methylation patterns in different pig breeds and tissues. Choi and colleagues reported the DNA methylome profiles of five different tissues [15]. Zhang and colleagues revealed the epigenetic mechanism of hypoxic adaptation in Tibetan and Yorkshire pigs [16]. Wang and Kadarmideen provided an epigenome-wide DNA methylation map of testis by a genome-wide DNA methylation analysis [17]. However, few studies have investigated the different epigenetic patterns between CHP and YP.

The main objective of this study was to explore the DNA methylation differences between CHP and YP by genome-wide DNA methylation analysis and then identify key genes and candidate epigenetic biomarkers associated with these differences of meat quality traits. We identified the differentially methylation regions (DMRs) and differentially methylation genes (DMGs) of CHP and YP to determine some of the important genomic regions and key genes associated with these phenotypic differences and providing new insights into the epigenetic mechanisms underlying the differences between the two pig breeds.

## Results

### Meat quality traits

Meat quality traits, including pH45min, pH24h, lightness (L*), redness (a*), and yellowness (b*), were assessed at 45 min and 24 h postmortem. Table 1 summarized the meat quality traits of the two breeds. Between the CHP and YP, the pH_45min_ (*P* = 7.78e-10), pH_24h_ (*P* = 1.24e-4), L*_45min_ (*P* = 1.95e-4), a*_45min_ (*P* = 1.06e-6), and b*_45min_ (*P* = 1.33e-3) of CHP were higher than those of YP.

**Table 1:** Summary of meat quality traits of the two breeds

Meat quality traits^a^	CHP (*n* = 20)	YP (*n* = 28)	*P*
pH_45min_	6.66 ± 0.12	6.22 ± 0.18	7.78e-10
pH_24h_	5.93 ± 0.51	5.57 ± 0.16	1.24e-4
L*_45min_	47.41 ± 2.58	42.58 ± 6.11	1.95e-4
a*_45min_	8.00 ± 1.79	5.22 ± 1.65	1.06e-6
b*_45min_	6.95 ± 0.75	5.25 ± 2.85	1.33e-3
L*_24h_	48.35 ± 3.77	50.94 ± 4.41	3.85e-2
a*_24h_	10.30 ± 2.78	9.11 ± 2.11	9.37e-2
b*_24h_	7.64 ± 1.16	7.12 ± 1.11	0.18

^a^Meat quality traits: measurements of meat quality traits, including muscle pH values, lightness (L*), redness (a*), and yellowness (b*) at 45 min and 24 h. *CHP* Chenghua pigs, *YP* Yorkshire pigs

### Summary of RRBS data

**Fig. 1:**
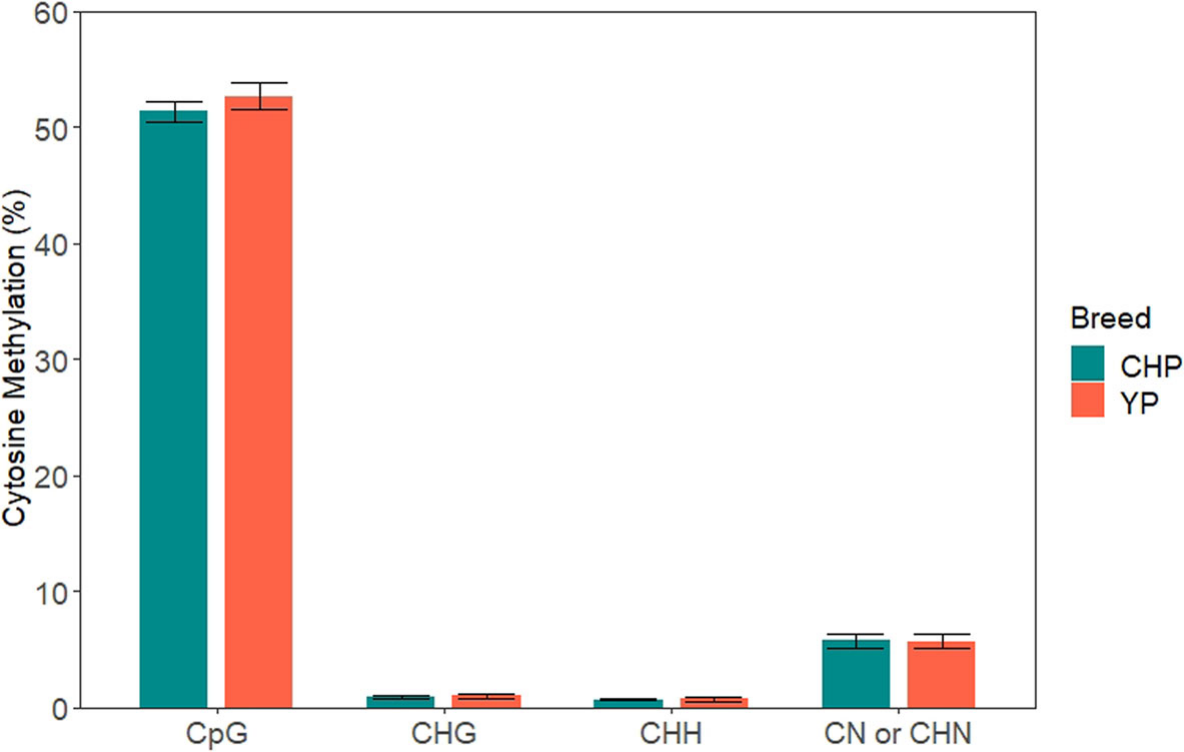
Comparison of the methylation level of CpG and non-CpG sites between CHP and YP. Non-CpG methylation was divided into CHG, CHH, CN, or CHN

Approximately 690.32Gb raw data was generated by RRBS from 48 muscle tissue samples of CHP and YP (approximately 14.38Gb raw data per individual). After quality control, approximately 523.72Gb clean data was obtained (approximately 10.91Gb clean data per individual). Besides, approximately 65% of the reads were mapped to the porcine reference genome (Table 2). Moreover, in all individuals, the density of normalized reads mapped to the proximal and distal regions of the chromosomes was higher than that of reads mapped to other regions. Overall methylated cytosines in the CpG/CHG/CHH (whereby H can be either A, T, or C) context were 51.39%/0.96%/0.7% in CHP and 52.68%/1.04%/0.78% in YP, respectively. Besides, C methylated in an unknown context like CN or CHN (whereby N can be either A, T, G, or C) was observed to be 5.8% in CHP and 5.77 in YP. Figure 1 shows CpG- and non-CpG-methylation sites (CHG, CHH, CN, or CHN) in muscle tissue of CHP and YP.

**Table 2:** Mapping results of reduced representation bisulfite sequencing (RRBS) data in the two breeds

Breeds	Mean raw data (Gb)	Mean clean data (Gb)	Mean BS Conversion Rate (%)	Mean mapping rate (%)
CHP	14.16	10.59	99.55	63.60
YP	14.54	11.14	99.40	65.61

*CHP* Chenghua pigs, *YP* Yorkshire pigs

### DMRs in the two groups

**Fig. 2:**
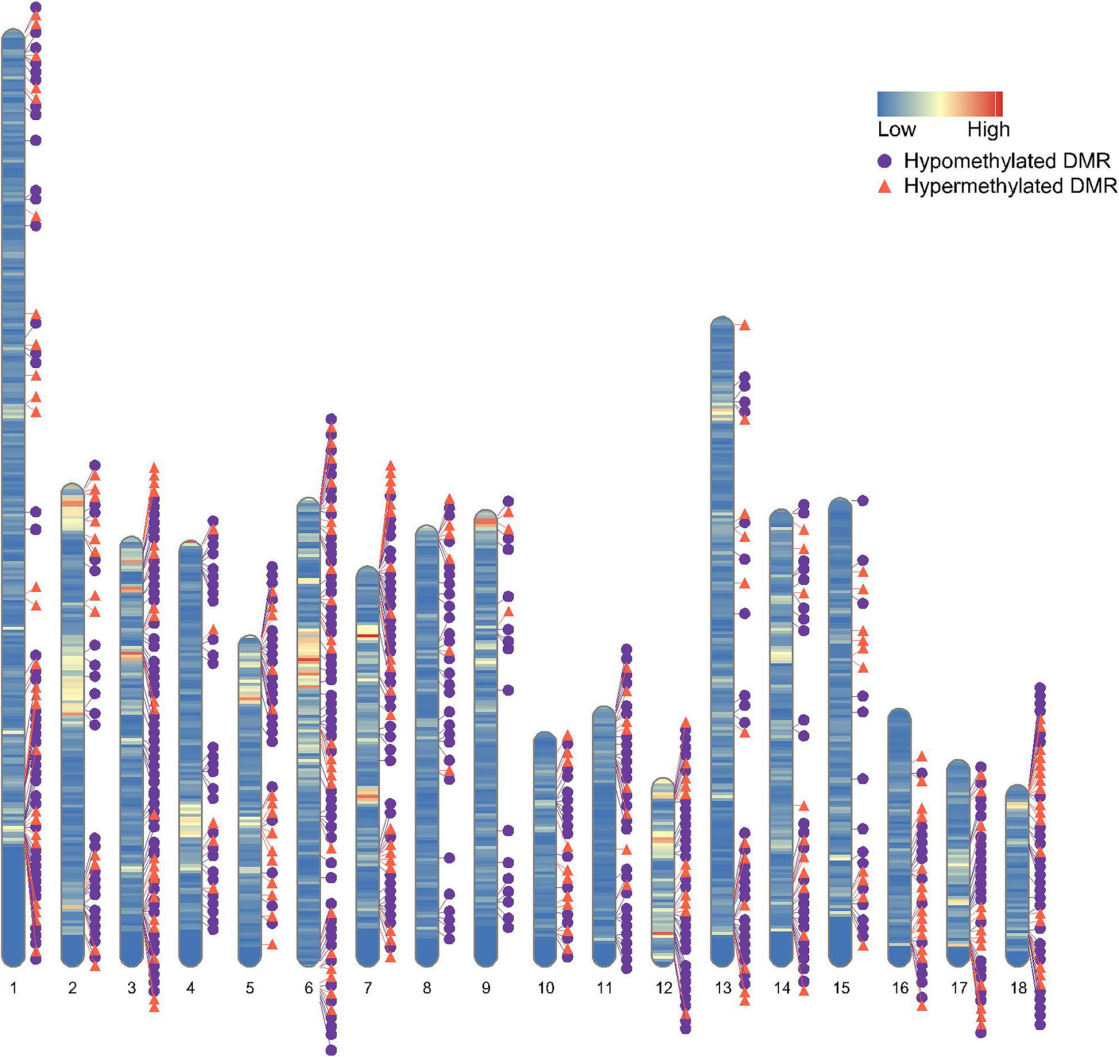
The distribution of differentially methylated regions (DMRs) throughout the whole genome in CHP vs. YP. The purple circle represents the hypomethylated DMRs. The orange triangle represents the hypermethylated DMRs. The color on a chromosome represents the gene density

Table 3 summarized the numbers of CpG sites and DMRs identified by CHP vs. YP comparison. A total of 2,416,211 CpG sites and 722 DMRs were identified by CHP vs. YP comparison (Fig. 2). Of these 2,416,211 CpG sites, the distribution of 2,416,211 CpG sites annotation within promoter, exon, intron, and intergenic regions was 28.31, 15.08, 36.01, and 20.60%, respectively. Additionally, percentages of 2,416,211 sites annotated within CpG islands, CpG island shores, and other regions were 48.93, 18.96, and 32.11%. However, of these 722 DMRs in CHP vs. YP group, 3.32% were overlapped with promoter regions, 12.33% with exons, 50.69% with introns, and 33.66% with intergenic regions (Table 3). Most DMRs were in introns regions, followed by intergenic, exons, and promoters. Furthermore, 12.19% DMRs in CHP vs. YP group were CpG island regions, 16.48% in the CpG shore region, and 71.33% in other regions. Of these DMRs, much fewer (32.55% in CHP vs. YP comparison) were hypermethylated in CHP (Table S1). Six key genomic regions were identified by the CHP vs. YP comparison (Table 4). These genomic regions contained multiple DMRs shared by the CHP vs. YP comparison and DMGs. The region on SSC1 (253.47–274.23 Mb) contained 34 DMRs and 18 DMGs. The region on SSC6 (148.71–169.49 Mb) contained 18 DMRs and 8 DMGs. The region on SSC7 (0.25–9.86 Mb) contained 26 DMRs and 6 DMGs. The region on SSC12 (43.06–61.49 Mb) contained 18 DMRs and 11 DMGs. The region on SSC14 (126.43–140.95 Mb) contained 17 DMRs and 9 DMGs. The region on SSC18 (49.17–54.54 Mb) contained 11 DMRs and 6 DMGs.

**Table 3:** Annotation of CpG sites and differential methylated regions (DMRs) in the pairwise comparison

Genetic features	Number	Annotated with gene^a^				Annotated within CpG^b^		
		Promoter	Exon	Intron	Intergenic	CpG island	CpG shore	Other regions
CpG sites	2,416,211	28.31%	15.08%	36.01%	20.60%	48.93%	18.96%	32.11
DMRs	722	3.32%	12.33%	50.69%	33.66%	12.19%	16.48%	71.33%

^a^Annotated with gene, the percentage of CpG sites or differential methylated regions that overlap with gene promoter, exon, intron, or intergenic; ^b^Annotated within CpG, the percentage of CpG sites or differential methylated regions that overlap with CpG island, CpG shore or other regions

**Fig. 3:**
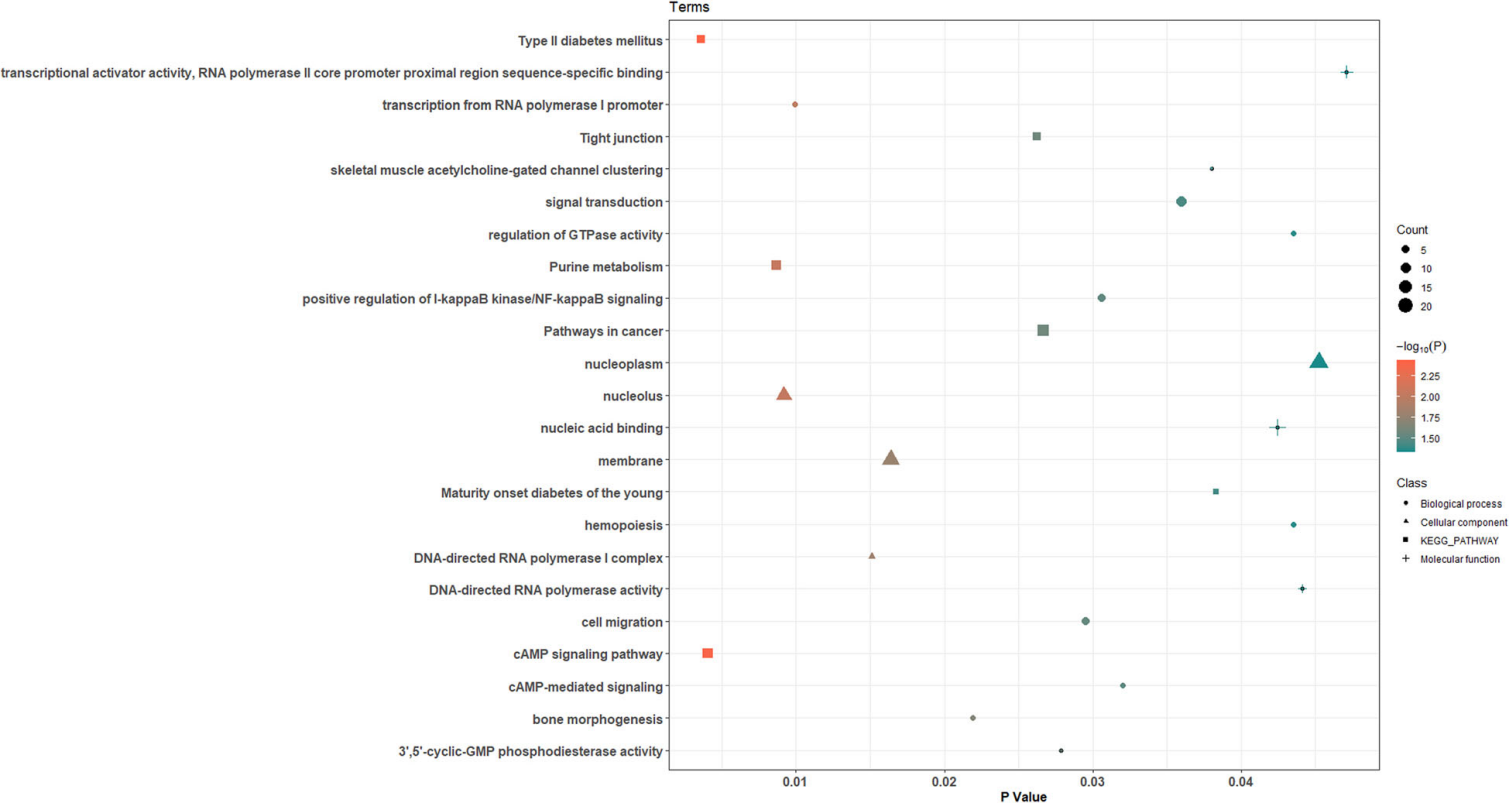
The bubble diagram of Gene Ontology (GO) and Kyoto Encyclopedia of Genes and Genomes (KEGG) pathway terms in CHP vs. YP group. The X-axis represents the *P* value of genes enriched in the corresponding GO and KEGG pathway terms. The Y-axis represents the GO and KEGG pathway terms to which the genes enriched. The shape of bubble represents the classification of GO and KEGG pathway terms. The color of bubble represents the log transformation of *P* value

**Table 4:** Six key genomic regions identified by the pairwise comparison

Genomic regions	Number of DMRs	Related DMGs^a^
SSC1:253.47–274.23 Mb	34	*SNX30, RGS3, ANAK, CDK5RAP2, ADGRD2, RABEPK, FAM102A, DNM1, GPR107, HMCN2, FUBP3, ABL1, FAM163B, NUP214, MED27, AK8, VAV2, OLFM1*
SSC6:148.71–169.49 Mb	18	*ROR1, NFIA, DAB1, CDCP2, AGBL4, RAD54L, PRFX1, ERI3*
SSC7:0.25–9.86 Mb	26	*EXOC2, GMDS, PSMG4, RPRF4B, CDYL, PHACTR1*
SSC12:43.06–61.49 Mb	18	*UTP6, KSR1, SLC13A2, ABR, NXN, WSCD1, ALOX15, CLDN7, MYH8, SLC47A1, RAI1*
SSC14:126.43–140.95 Mb	17	*GFRA1, PLPP4, BUB3, DHX32, ADAM12, PTPRE, MGMT, TCERG1L, CFAP46*
SSC18:49.17–54.54 Mb	11	*TNS3, CCDC201, ADCY1, NPC1L1, GCK, CAMK2B*

^a^Related DMGs: Based on the Ensemble database (http://asia.ensembl.org/Sus_scrofa/Info/Index). *DMRs* differentially methylated regions, *DMGs* differentially methylated genes, *SSC Sus scrofa* chromosome

### DMGs identified according to DMRs and functional annotation of DMGs

We annotated 466 DMGs from DMRs identified by comparing CHP vs. YP. Besides, 149 DMGs exhibited higher levels of DNA methylation in CHP than in YP (Table S2), while 317 DMGs exhibited lower levels of DNA methylation in CHP than in YP (Table S3).

The main GO terms enriched in 466 DMGs that were identified by CHP vs. YP comparison (Fig. 3 and Table S4) and that might be related to nucleolus (*P* = 9.18e-3), transcription from RNA polymerase I promoter (*P* = 9.94e-3), DNA-directed RNA polymerase I complex (*P* = 1.15e-2), and membrane (*P* = 1.64e-2), while the KEGG pathways included Type II diabetes mellitus (*P* = 3.62e-3), cAMP signaling pathway (*P* = 4.05e-3), and Purine metabolism (*P* = 8.71e-3). According to the functions of DMGs, we identified 8 DMGs (*ADCY1*, *AGBL4*, *EXOC2*, *FUBP3, PAPPA2*, *PIK3R1*, *MGMT,* and *MYH8*) which were possibly related to the difference in appearance, meat quality, disease resistance, reproductive capacity, and adaptability between CHP and YP (Table 5).

**Table 5:** The summary of 8 key DMGs identified by the pairwise comparison

DMGs	SSC	Start^a^	End^a^	DMR	Function
*ADCY1*	18	50,046,575	50,143,771	SSC18:50056501–50,057,000	Related to pigmentation
*AGBL4*	6	161,952,983	163,216,257	SSC6:163155001–163,155,500	Associated with skeletal formation
*EXOC2*	7	195,081	341,454	SSC7:251501–252,000	Related to the tanning ability
*FUBP3*	1	270,652,398	270,705,033	SSC1:270700001–270,700,500	Associated with loin eye area
*PAPPA2*	9	118,364,592	118,635,969	SSC9:118601501–118,602,000	Plays an important role in regulation of IGF-I bioavailability
*PIK3R1*	16	46,434,873	46,523,609	SSC16:46495001–46,495,500	Be essential for myogenic differentiation
*MGMT*	14	138,499,161	138,779,938	SSC14:138646501–138,647,000	Involved in a wide spectrum of human cancers
*MYH8*	12	55,134,844	55,167,749	SSC12:55148501–55,149,000	Belonged to the myosin heavy chain gene family

^a^Based on the Ensemble database (http://asia.ensembl.org/Sus_scrofa/Info/Index). *DMRs* differentially methylated regions, *DMGs* differentially methylated genes, *SSC Sus scrofa* chromosome

## Discussion

In this study, we found that there were differences in DNA methylation between CHP and YP. The methylation patterns of CHP may help to explain the epigenetic regulation mechanisms of traits.

Bisulfite sequencing is an ideal and practical technique for studying epigenetic modifications of different species and tissues [18], especially DNA methylation, which can detect the DNA methylation level at each base position of the whole genome. However, genome-wide DNA methylation sequencing with high coverage of the whole genome is required to accurately assess the methylation levels at each base position. Thus, RRBS was used in this study because of its high coverage, small data requirement, low cost, and simple operation. Compared to other studies in pigs [16, 17, 19], this study used a larger population size. Therefore, RRBS is suitable for detecting DNA methylation differences among breeds in this study.

We observed several interesting GO terms and KEGG pathways associated with muscle metabolism and development. The KEGG pathways of Type II diabetes mellitus (enriched with *MAPK10*, *PRKCE*, *GCK*, *MTOR*, *PIK3R1*), cAMP signaling pathway (enriched with *ADCY1*, *ACOX3*, *ADCY5*, *ARAP3*, *PIK3R1*, *MAPK10*, *GRIN2B, VIPR2,* and *VAV2*), the GO terms of skeletal muscle acetylcholine-gated channel clustering (enriched with *COLQ* and *DNAJA3*) and the cAMP-mediated signaling (enriched with *ADCY1, ADCY5, and KSR1*) were identified in CHP vs. YP. As a major metabolic tissue, metabolic-related pathways and GO terms, including Type II diabetes mellitus, cAMP signaling pathway, skeletal muscle acetylcholine-gated channel clustering, and cAMP-mediated signaling was enriched in this study. The results indicated that DMGs associated with these metabolic processes show significant differences between CHP and YP. The pork pH has an important relationship with muscle metabolism. In this study, the pH_45min_ (*P* = 7.78e-10) and pH_24h_ (*P* = 1.24e-4) of CHP were higher than those of YP. Therefore, DMGs involved in muscle metabolism were identified in CHP and YP, which suggested that the difference of pH between the two breeds may be influenced by these pathways and related genes.

cAMP signaling pathway is a crucial pathway which regulates pivotal physiologic processes including metabolism, secretion, calcium homeostasis, muscle contraction, cell fate, and gene transcription. In this study, 9 DMGs are enriched in the cAMP signaling pathway. Two of these DMGs, including *ADCY1* and *PIK3R1*, are related to melanoma metastasis. Previous studies have shown that knockdown of *ADCY1* gene leads to decreased intracellular cAMP and subsequently inhibits PKA activity, and phospho- cAMP-responsive element binding protein (CREB) and microphthalmia-associated transcription factor (MITF) levels were significantly downregulated after inactivation of PKA [20]. Furthermore, CREB and MITF have been implicated in melanoma tumor growth and metastasis [21–23]. Besides, the *ADCY1* gene was identified as a key candidate gene involved in melanoma metastasis [24]. There is an important link between pigmentation and melanoma. This result suggests that *ADCY1* gene may affect pigmentation through cAMP. The PI3K protein, encoded by *PIK3R1* gene, is a key protein involved in the PI3K/AKT signaling pathway, which is essential for myogenic differentiation [25] and regulates cell survival, growth, differentiation, glucose transport, and utilization [26]. Therefore, the high levels of methylation of *ADCY1* and *PIK3R1* in CHP may trigger changes in their expression, potentially leading to different meat color traits between CHP and YP.

Notably, some other key DMGs, including *AGBL4*, *EXOC2*, *FUBP3, PAPPA2*, *MGMT, and MYH8* were found in this study. The *AGBL4* gene was regarded as a candidate gene associated with the heterotic quantitative trait in beef cattle [27]. A genome-wide association study (GWAS) suggested that one SNP (rs12210050) in *EXOC2* was related to the tanning ability of Europeans [28]. A previous study demonstrated that the *FUBP3* gene was associated with the skeletal formation in Duroc population [29]. Furthermore, the *FUBP3* gene was identified as a candidate gene associated with the loin eye area in pigs [30]. The *PAPPA2* gene encodes pregnancy-associated plasma protein A2 (PAPPA2) which plays an important role in the regulation of IGF-I bioavailability [31]. It is a metalloproteinase that can specifically clew IGFBP-3 and IGFBP-5, thereby releasing IGF-I from its ternary complex, enabling it to bind to IGF-I receptors on the cell surface, initiating growth-promoting activity [32]. Besides, in genome-wide association analysis, *PAPPA2* and its related gene, *PAPPA*, were identified as common genetic variants associated with adult stature in the general population [33]. The *MGMT* gene is a DNA repair gene responsible for removing alkylation adducts from the O6-position of guanine in DNA. The promoter CpG island hypermethylation associated gene silencing of *MGMT* is involved in a wide spectrum of human cancers, including glioblastoma [34], gastric [35], colon [36], and ovarian [37]. The *MYH8* gene belonged to the myosin heavy chain gene family that share the common features of ATP hydrolysis, actin binding, and potential for kinetic energy transduction [38]. Moreover, the MYH8 myosin is re-expressed during muscle regeneration and is deemed as a specific marker of regenerating fibers in the pathologic skeletal muscle [39, 40].

## Conclusion

This study performed epigenome-wide DNA methylation analysis using RRBS data generated for muscle tissues of 48 pigs. CHP vs. YP revealed 722 DMRs and 466 DMGs based on these DMRs. Besides, 6 key genomic regions and 8 key DMGs, which might be related to phenotypic differences between CHP and YP, were identified according to the further functional analysis. Our finding may help to further understand the epigenetic mechanisms of phenotype traits and have reference significance for the local pork industry.

## Methods

### Animals and measurements of meat quality

Totals of 48 healthy pigs were used in this study from two pig breeds, including CHP (*n* = 20) and YP (*n* = 28). These pigs were maintained in a similar environment to avoid the effects of other confounders. There are 10 males and 10 females in the Chenghua pigs, and there are 20 males and 8 females in the Yorkshire pigs. Each population contains a certain number of males and females. In addition, a large sample size was used to reduce the influence of confounders. Animals were slaughtered at a commercial slaughterhouse when they reached the slaughter weight of 105 kg. Subsequently, muscle pH values were measured at 45 min and 24 h postmortem using a portable pH meter (model 720A; Orion Research Inc., Boston, MA, USA). Meat color, including lightness (L*), redness (a*), and yellowness (b*) were assessed at 45 min and 24 h postmortem on the longissimus dorsi muscle using a Minolta CR-300 colorimeter (Minolta Camera, Osaka, Japan). After slaughter, tissue samples from muscle were collected from each breed for DNA isolation. Tissue samples were frozen in liquid nitrogen and stored at − 20 °C until analysis. All experimental procedures and sample collection were approved by the Institutional Animal Care and Use Committee of the College of Animal Science and Technology of Sichuan Agricultural University, Sichuan, China, under permit No. DKY-B20121403.

### Library construction

Briefly, genomic DNA was isolated from flash frozen muscular tissue. Then, the construction of RRBS libraries and paired-end sequencing using Illumina HisSeq analyzer was performed at Novogene technology co., LTD (Beijing, China). Raw sequencing data were processed by an Illumina base-calling pipeline. Genomic DNA was digested with *Msp*I enzyme at 37 °C for 16 h. The DNA fragments after enzyme digestion were repaired at the end, and the sequencing adapters with all cytosine methylated were attached. The inserted DNA fragments with the length ranging from 40 to 220 bp were selected for glue cutting. Then, Bisulfite conversion was carried out. After that, the unmethylated C was changed to U (after PCR amplification to T), while the methylated C remained unchanged. Finally, PCR amplification was carried out to obtain the final DNA library. Clean reads were obtained from the raw data after removing reads containing adaptor sequences, unknown, or low-quality bases. The process of quality control was carried out using Trimmomatic software [41]. Quality control was adopted to access the high data quality by (1) removing low-quality reads using a sliding window method (SLIDINGWINDOW: 4:15); (2) removing reads including adaptor sequences (ILLUMINACLIP: adapter.fa: 2:30:7:1: true); (3) removing reads with tail quality lower than 3 or with unknown bases (TRAILING: 3).

### Data analysis

Clean reads were aligned to the pig reference genome (Sscrofa11.1) using Bismark v0.22.1 [42]. This progress includes three steps: genome preparation, alignment using Bowtie 2 v2.3.5.1 [43], and methylation extractor. Bismark methylation extractor outputs read coverage and methylation percentage of detected methylated or unmethylated reads at one genomic position. The R package methyKit v1.14.2 [44] was used to identify DMRs (window size = 500 bp, qvalue< 0.01, methylation difference > =0.25) based on the Bismark coverage file. The R package Rldeogram v0.2.2 [45] was used to visualize the distribution of DMR. The DMGs within DMRs were annotated using the Ensemble database. The R package genomation v1.20 [46] was used to perform annotation of DMRs. The porcine RefSeq and CpG island database (Sscrofa11.1/susScr11) for annotations were derived from the UCSC website (http://genome.ucsc.edu/cgi-bin/hgTables).

### Enrichment analysis

Significant GO terms and KEGG pathways were selected after filtering with *P* < 0.01. R package ggplot2 v3.3.2 was used to visualize the significant GO terms and KEGG pathways for the DMGs associated with DMRs.

## Supplementary Information

**Additional file 1: Table S1**. Summary of hypermethylation regions in CHP vs. YP.

**Additional file 2: Table S2**. Summary of hypomethylation regions in CHP vs. YP.

**Additional file 3: Table S3**. Summary of hypermethylation genes in CHP vs. YP.

**Additional file 4: Table S4**. Summary of hypomethylation genes in CHP vs. YP.

**Additional file 5: Table S5**. Summary of Gene Ontology (GO) enrichment and Kyoto Encyclopedia of Genes and Genomes (KEGG) pathway analysis in CHP vs. YP.

## Data Availability

The datasets generated during and/or analysed during the current study are available in the Figshare repository (https://figshare.com/) with the DOI: 10.6084/m9.figshare.14684382.v1

## Abbreviations

CHP: Chenghua pig
YP: Yorkshire pig
RRBS: Reduced representation bisulfite sequencing
DMRs: Differentially methylated regions
DMGs: Differentially methylated genes
SSC: *Sus scrofa* chromosome
GO: Gene Ontology
KEGG: Kyoto Encyclopedia of Genes and Genomes
CpG: Cytosine and guanine dinucleotide
GWAS: Genome-wide association study
